# ProteinBERT: a universal deep-learning model of protein sequence and function

**DOI:** 10.1093/bioinformatics/btac020

**Published:** 2022-02-10

**Authors:** Nadav Brandes, Dan Ofer, Yam Peleg, Nadav Rappoport, Michal Linial

**Affiliations:** School of Computer Science and Engineering, The Hebrew University of Jerusalem, Jerusalem 9190401, Israel; Department of Biological Chemistry, The Alexander Silberman Institute of Life Sciences, The Hebrew University of Jerusalem, Jerusalem 9190401, Israel; Deep Trading Ltd., Haifa 3508401, Israel; Department of Software and Information Systems Engineering, Faculty of Engineering Sciences, Ben-Gurion University of the Negev, Beer Sheva 8410501, Israel; Department of Biological Chemistry, The Alexander Silberman Institute of Life Sciences, The Hebrew University of Jerusalem, Jerusalem 9190401, Israel

## Abstract

**Summary:**

Self-supervised deep language modeling has shown unprecedented success across natural language tasks, and has recently been repurposed to biological sequences. However, existing models and pretraining methods are designed and optimized for text analysis. We introduce ProteinBERT, a deep language model specifically designed for proteins. Our pretraining scheme combines language modeling with a novel task of Gene Ontology (GO) annotation prediction. We introduce novel architectural elements that make the model highly efficient and flexible to long sequences. The architecture of ProteinBERT consists of both local and global representations, allowing end-to-end processing of these types of inputs and outputs. ProteinBERT obtains near state-of-the-art performance, and sometimes exceeds it, on multiple benchmarks covering diverse protein properties (including protein structure, post-translational modifications and biophysical attributes), despite using a far smaller and faster model than competing deep-learning methods. Overall, ProteinBERT provides an efficient framework for rapidly training protein predictors, even with limited labeled data.

**Availability and implementation:**

Code and pretrained model weights are available at https://github.com/nadavbra/protein_bert.

**Supplementary information:**

Supplementary data are available at *Bioinformatics* online.

## 1 Background

Proteins are nature’s ultimate machines, found across the entire tree of life. While knowledge of protein sequences is accumulating exponentially, understanding their functions remains one of the greatest scientific challenges of our time, with numerous implications to human health. Protein sequences can be viewed as strings of amino-acid letters. As such, machine-learning methods developed for natural language and other sequences are a natural fit to predictive protein tasks (Ofer *et al.*, 2021).

Modern deep neural network architectures specifically designed for sequences [such as BERT (Devlin *et al.*, 2018; Vaswani *et al.*, 2017)], combined with pretraining on massive datasets, have led to a revolution in automated text analysis (Radford *et al.*, 2019). The attention-based Transformer architecture in particular has shown astounding performance over a wide range of benchmarks across many domains (Brown *et al.*, 2020; Keskar *et al.*, 2019).

At the heart of these successes are self-supervised and transfer learning. According to the transfer-learning paradigm, a model is first pre-trained on one task, and then fine-tuned on other downstream tasks of interest (Do and Ng, 2005; Pan and Yang, 2010; Raffel *et al.*, 2019). Assuming that the pretraining and downstream tasks are somehow related (e.g. both require understanding texts in the same language), pretraining can help the model learn useful representations for the downstream tasks. In self-supervised pretraining, labels are automatically generated, allowing models to learn from enormous, unlabeled datasets (Chen *et al.*, 2020). A common example of self-supervised learning is language modeling, where a model (typically a deep neural network) learns language structure by filling missing parts in a text (which have been hidden with a special mask token) or reconstructing corrupted text (Howard and Ruder, 2018; Radford *et al.*, 2018). Fine-tuning, on the other hand, is typically supervised and requires labeled data. The transfer-learning paradigm has allowed predictive models to achieve substantial performance gains across numerous benchmarks, especially in tasks where labeled data is scarce (Thrun, 1996; Wang *et al.*, 2019).

Most sequence-based language models [e.g. BERT (Devlin *et al.*, 2018), ULMFiT (Howard and Ruder, 2018), XLNet (Yang *et al.*, 2019), ELECTRA (Clark *et al.*, 2020)] have been designed for processing natural languages (with a bias toward English). Thus, their architectures and pretraining tasks may not be optimal for proteins, which, despite many structural similarities, have different properties from human language (Ofer *et al.*, 2021; Strait and Dewey, 1996). Most notably, proteins do not have clear-cut multi-letter building blocks (such as words and sentences). Moreover, proteins are more variable in length than sentences, and show many interactions between distant positions (due to their 3D structure). To this day, protein research is still dominated by classical sequence-similarity methods [such as BLAST (Altschul *et al.*, 1990) and hidden Markov models (Finn *et al.*, 2014)], in contrast to domains such as computer vision which have become dominated by deep learning. A few recent studies have pretrained deep neural language models on protein sequences [e.g. ESM (Rives *et al.*, 2021), TAPE-Transformer (Rao *et al.*, 2019), ProtTrans (Elnaggar *et al.*, 2021), UDSMProt (Strodthoff *et al.*, 2020), UniRep (Alley *et al.*, 2019)] (Heinzinger *et al.*, 2019; Madani *et al.*, 2020; Nambiar *et al.*, 2020). Such works usually import existing architectures and tasks from the natural language domain, without taking advantage of the unique characteristics of proteins. A handful of works have implemented protein-centric pretraining tasks, with mixed success and without changing the underlying architecture (Rao *et al.*, 2021; Sturmfels *et al.*, 2020; Yang *et al.*, 2020).

Here, we present ProteinBert, a new deep-learning model designed for protein sequences. We improve upon the classic Transformer/BERT architecture, and introduce a novel pretraining task of predicting protein functions. We pretrained ProteinBert on ∼106M proteins (representing the entire known protein space) on two simultaneous tasks. The first task is bidirectional language modeling of protein sequences. The second task is Gene Ontology (GO) annotation prediction, which captures diverse protein functions (Ashburner *et al.*, 2000). GO annotations are a manually curated set of ∼45K terms defined at the whole-protein level, covering the entire protein space across all organisms. They cover molecular functions, biological processes and subcellular locations. Unlike classic Transformers, ProteinBERT separates local (character level) and global (whole sequence level) representations (as well as inputs and outputs), thereby supporting multitasking of both local and global tasks in a principled way. While ProteinBERT is considerably smaller and faster than existing models, it approaches or exceeds state-of-the-art performance on a diverse set of benchmarks.

## 2 Materials and methods

### 2.1 Data

#### 2.1.1 Protein dataset for pretraining

ProteinBERT was pretrained on ∼106M proteins derived from UniProtKB/UniRef90, covering the entire tree of life (Boutet *et al.*, 2016; Suzek *et al.*, 2007). UniRef90 provides a non-redundant set of protein clusters sharing at least 90% sequence identity. Each cluster is represented by a single representative protein, ensuring a relatively homogenous coverage of the protein space. For each protein, we extracted its amino-acid sequence and associated GO annotations (according to UniProtKB). We considered only the 8943 most frequent GO annotations that occurred at least 100 times in UniRef90. Of the ∼106M UniRef90 proteins, ∼46M had at least one of the 8943 considered annotations (with ∼2.3 annotations per protein, on average across the ∼46M proteins).

#### 2.1.2 Protein benchmarks

To evaluate ProteinBERT, we tested it on nine benchmarks representing all major facets of protein research, covering protein function, structure, post-translational modifications and biophysical properties (Table 1). Labels in these benchmarks are either local (e.g. post-translational modifications) or global (e.g. remote homology), and they are either continuous (e.g. protein stability), binary (e.g. signal peptide) or categorical (e.g. secondary structure). Notably, in local benchmarks, the number of training samples is much greater than the number of protein sequences, as target labels are per residue.

**Table 1.** Protein benchmarks

Topic	Benchmark	Target type^a^	Resolution	# Training sequences	Source
Protein structure	Secondary structure	Categorical (3)	Local	8,678	(Moult *et al.*, 2018; Rao *et al.,* 2019)
	Disorder	Binary	Local	8,678	(Moult *et al.,* 2018)
	Remote homology	Categorical (1,195)	Global	12,312	(Andreeva *et al.,* 2014, 2020; Rao *et al.,* 2019)
	Fold classes	Categorical (7)	Global	15,680	(Andreeva *et al.,* 2014, 2020)
Post-translational modifications	Signal peptide	Binary	Global	16,606	(Armenteros *et al.,* 2019)
	Major PTMs	Binary	Local	43,356	(Hornbeck *et al.,* 2015)
	Neuropeptide cleavage	Binary	Local	2,727	(Ofer and Linial 2014, 2015; Brandes *et al.,* 2016)
Biophysical properties	Fluorescence	Continuous	Global	21,446	(Sarkisyan *et al.*, 2016; Rao *et al.,* 2019)
	Stability	Continuous	Global	53,679	(Rocklin *et al.,* 2017; Rao *et al.,* 2019)

a For categorical targets, the number of classes appears in parentheses.

Four out of nine benchmarks (secondary structure, remote homology, fluorescence and stability) were taken from TAPE (Tasks Assessing Protein Embeddings), a standardized set of benchmarks for evaluating protein sequence models (Rao *et al.*, 2019). The ‘contact prediction’ task from TAPE was not included in this analysis, as it does not fit the model’s output. Specifically ProteinBERT outputs global and per-position outputs, but not pairwise outputs. In addition, we introduce five new benchmarks (see Supplementary Methods). Comparisons between ProteinBERT and other models were carried out on the four TAPE benchmarks. Internal evaluations were also carried out on the five new benchmarks (see Section 3).

### 2.2 Sequence and annotation encoding

Protein sequences were encoded as sequences of integer tokens. We used 26 unique tokens representing the 20 standard amino acids, selenocysteine (*U*), an undefined amino-acid (*X*), another amino acid (*OTHER*) and 3 additional tokens (*START*, *END* and *PAD*). For each sequence, *START* and *END* tokens were added before the first amino acid and after the last amino acid, respectively. The *PAD* token was added to pad sequences shorter than the sequence length chosen for the minibatch.

The architecture of ProteinBERT (like most deep learning models) dictates that each minibatch has a fixed sequence length. We included the *START* and *END* tokens to help the model interpret proteins that are longer than the chosen sequence length. When encoding a protein longer than the chosen sequence length, we selected a random subsequence of the protein, leaving out at least one of its two ends. The absence of the START or END token allowed the model to recognize that it only received part of a sequence.

GO annotations of every sequence were encoded as a binary vector of fixed size (8943), where all entries are zeros except those corresponding to GO annotation associated with the protein. When no information about GO annotations is provided to the model (e.g. during fine-tuning and evaluation on the benchmarks), the vector is set to all zeros.

### 2.3 Self-supervised pretraining on protein sequences and annotations

To learn protein representations, ProteinBERT was pretrained on protein sequences and GO annotations extracted from UniRef90. The model received corrupted inputs (protein sequences and GO annotations) and had to recover the uncorrupted data. The corruption of protein sequences was performed by randomly replacing tokens with 5% probability (i.e. keeping the original token with 95% probability, or replacing it with a uniformly selected random token with 5% probability). Input GO annotations were corrupted by randomly removing existing annotations with 25% probability, and adding random false annotations with probability of 0.01% for each annotation not associated with the protein. For 50% of the processed proteins, we removed all input annotations altogether (i.e. giving an all-zero input vector), to force the model to predict GO annotations from sequence alone (as was the case in all tested benchmarks). In summary, the described pretraining is a dual task, where the model has to recover both the protein sequence and its known GO annotations. The latter task is relevant to numerous domains of protein research, given the wide range of functions covered by GO terms.

To avoid leakage of information from GO annotations of proteins in the tested benchmarks (Table 1), we removed the GO annotations of any protein with at least 40% sequence similarity to any record in the benchmark test sets [using BLASTP with default parameters (Altschul *et al.*, 1997)], thereby making sure that ProteinBERT did not get additional information on the test-set proteins other than their sequences. Notably, 40% sequence similarity (as defined by BLAST) captures even lower percentages of sequence identity. Of the ∼106M proteins used for pretraining, ∼600K such sequences were identified.

The loss function minimized by ProteinBERT during pretraining was a sum of the categorical cross-entropy over the protein sequences and the binary cross-entropy over the GO annotations, namely $L=-\sum_{i=1}^{l}\log\left({\hat{S}}_{i,S_{i}}\right)-\sum_{j=1}^{8943}\left(A_{j}\cdot \log\left({\hat{A}}_{j}\right)+\left({1-A}_{j}\right)\cdot \log\left(1-{\hat{A}}_{j}\right)\right)$, where l is the sequence length, $S_{i}\in \{1,\ldots ,26\}$ is the sequence’s true token at position i, ${\hat{S}}_{i,k}\in [0,1]$ is the predicted probability that position i has the token k (for any k∈{1,…,26}), $A_{j}\in \{0,1\}$ is the true indicator for annotation j (for any j∈{1,…8943}), and ${\hat{A}}_{j}\in [0,1]$ is the predicted probability that the protein has annotation j.

An important feature of ProteinBERT is sequence length flexibility. To avoid the risk of overfitting the model to a specific constant length, we periodically (every 15 min of training) switched the encoding length of protein sequences, using lengths of 128, 512 or 1024 tokens.

Pretraining speed on a single GPU (Nvidia Quadro RTX 5000) was 280 protein records per second. We trained the model for 28 days over ∼670M records (i.e. ∼6.4 iterations over the entire training dataset of ∼106M records). The trained model weights are publicly available along with our code (see below).

### 2.4 Supervised fine-tuning on protein benchmarks

Following pretraining, we fine-tuned and evaluated the model on a diverse set of benchmarks (Table 1). For all benchmarks, ProteinBERT was initialized from the same pretrained state and fine-tuned through the same protocol. Initially, all layers of the pretrained model were frozen, and only a newly added fully connected layer was allowed to train for up to 40 epochs. Next, we unfroze all the layers and trained the model for up to 40 additional epochs. Finally, we trained the model for one final epoch of a larger sequence length (see Supplementary Methods). Throughout all epochs, we reduced the learning rate on plateau and applied early stopping based on an independent validation set. Model evaluation was then performed over a held-out test set. No information about GO annotations was exploited throughout the entire fine-tuning and benchmark evaluation process (i.e. the GO-annotation input was always a constant all-zero vector). The entire fine-tuning procedure took ∼14 min on a single GPU (on average across the nine benchmarks).

### 2.5 Deep-learning architecture

**Fig. 1.**
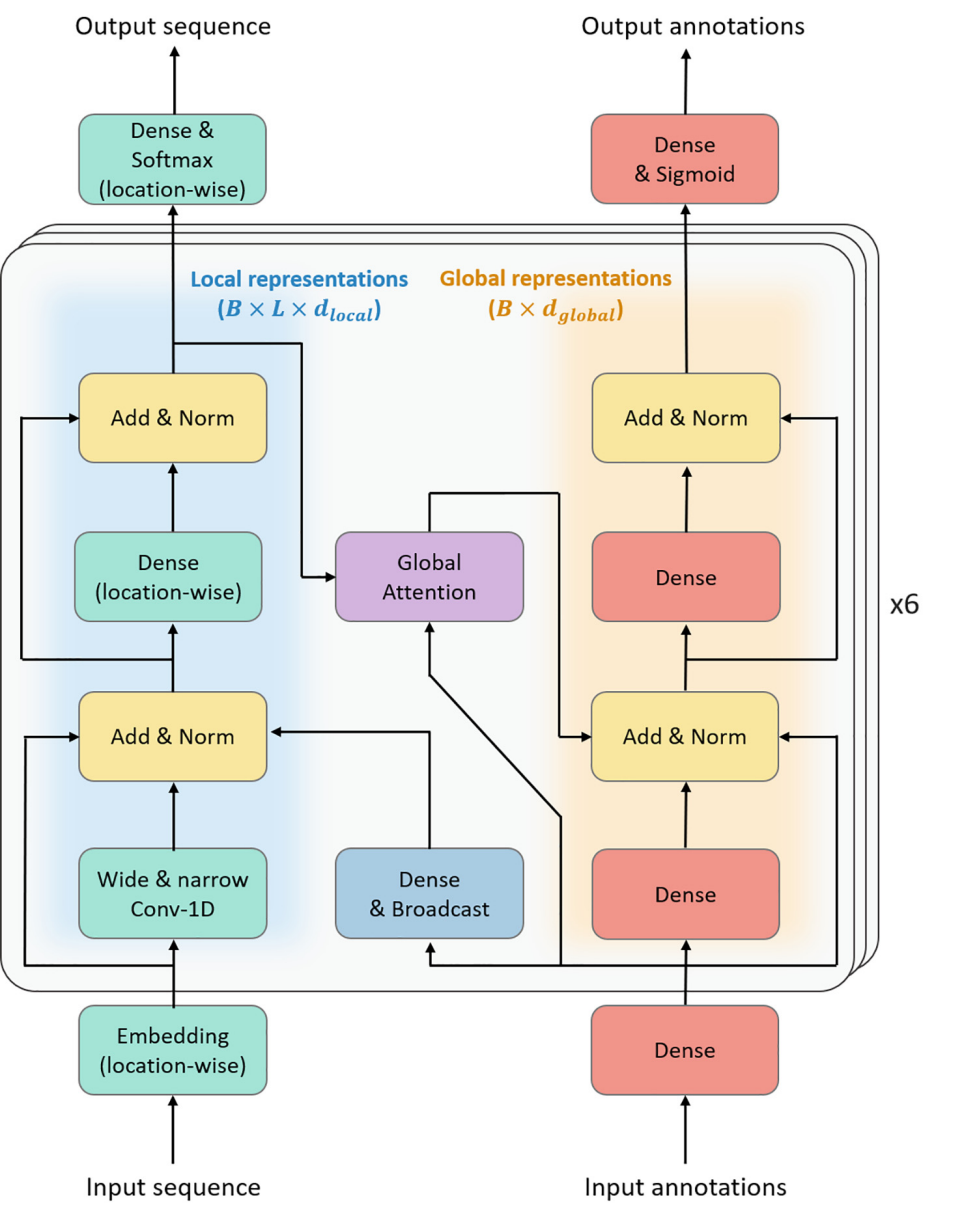
The ProteinBERT architecture. ProteinBERT’s architecture is inspired by BERT. Unlike standard Transformers, ProteinBERT supports both local (sequential) and global data. The model consists of six transformer-like blocks manipulating local (left side) and global (right side) representations. Each such block manipulates these representations by fully connected and convolutional layers (in the case of local representations), with skip connections and normalization layers between them. The local representations affect the global representations through a global attention layer, and the global representations affect the local representations through a broadcast fully connected layer

While inspired by BERT (Devlin *et al.*, 2018), the architecture of ProteinBERT is different and includes several innovations. ProteinBERT is a type of a denoising autoencoder (Fig. 1). The two inputs (and outputs) of ProteinBERT are (i) protein sequences (encoded as amino-acid token sequences) and (ii) GO annotations (encoded as fixed-size binary vectors).

The model architecture consists of two almost parallel paths: one for local representations and the other for global representations (Fig. 1). The local representations are 3D tensors of shape $B\times L\times d_{\mathrm{local}}$ where B is the batch size, L is the minibatch sequence length, and $d_{\mathrm{local}}$ is the number of channels for the local representations (we used $d_{\mathrm{local}}=128$). The global representations are 2D tensors of shape $B\times d_{\mathrm{global}}$ (using $d_{\mathrm{global}}=512$). In the first layers of the model, the input sequences are transformed into the local-representation 3D tensor by an embedding layer with $d_{\mathrm{local}}$ output features (which is applied independently and identically position-wise), and the input annotations are transformed into the global-representation 2D tensor by a fully connected layer with $d_{\mathrm{global}}$ output features.

The local and global representations are processed by a series of six transformer-like blocks with skip connections and layer normalizations between their hidden layers. Within each block, the local representation is transformed first by 1D convolutional layers, and then by a (location-wise) fully connected layer. To allow the local representations at each position to be based on other positions at both short and remote proximity, we used both a narrow (without dilation) and a wide (with dilation rate of 5) convolutional layer. Both types of convolution layers have a kernel size of 9 and stride size of 1. Accordingly, each narrow layer has a receptive field of 9 and each wide layer has a receptive field of 41 over the previous layer, meaning that the 6th block has a receptive field of 241 over the input sequence. The global representations, on the other hand, are transformed by two simple fully connected layers per block (with normalizations between them). All the hidden fully connected and convolutional layers of the model use GELU (Gaussian Error Linear Unit) activations (Hendrycks and Gimpel, 2016).

The only information flow between the local and global representations occurs through broadcast fully connected layers (from the global to the local representations) and global attention layers (from the local to the global representations). The broadcast layers are fully connected layers that transform the $d_{\mathrm{global}}$ features of the global representation into $d_{\mathrm{local}}$ features of the local representations, and then replicate that representation across each of the L sequence positions.

The global attention layer, inspired by self-attention (Vaswani *et al.*, 2017), is of linear (rather than quadratic) complexity. While self-attention takes an input sequence and outputs another sequence by allowing each position to attend to each other position, global attention takes as input both a sequence and a global fixed-size vector and outputs a global fixed-size vector created by attending to each of the local input positions according to the global input vector. Formally, a single-head global attention layer takes as inputs a global representation vector $x\in R^{d_{\mathrm{global}}}$ and local representation vectors across L positions, $s_{1},\ldots ,s_{L}\in R^{d_{\mathrm{local}}}$, and outputs a global output $y\in R^{d_{\mathrm{value}}}$. Similar to self-attention, the output is calculated by $y=\sum_{i=1}^{L}z_{i}v_{i}$ where $v_{i}\in R^{d_{\mathrm{value}}}$ is the value associated with each position i∈{1,…L} and $z_{i}\in [0,1]$ is the amount of attention allocated to that position (satisfying $z_{1}+\cdots +z_{L}=1$). Like in self-attention, the value associated with each position is calculated by $v_{i}=\sigma \left(W_{v}s_{i}\right)$, using a parameter matrix $W_{v}\in R^{d_{\mathrm{value}}\times d_{\mathrm{local}}}$ and an activation function σ (we chose GELU). Attention values are calculated by $z_{1},\ldots ,z_{L}=so\mathrm{ftmax}{\left\{\frac{〈q,k_{i}〉}{\sqrt{d_{\mathrm{key}}}}\right\}}_{i=1}^{L}$, based on query and key vectors $q,k_{i}\in R^{d_{\mathrm{key}}}$. Notice that while the key vectors $k_{1},\ldots ,k_{L}$ are specific to each position, the query vector q is global. Like in self-attention, the key vectors are calculated by $k_{i}=\tanh\left(W_{k}s_{i}\right)$, using a second parameter matrix $W_{k}\in R^{d_{\mathrm{key}}\times d_{\mathrm{local}}}$. The global query vector is calculated by $q=\tanh\left(W_{q}x\right)$, using a third parameter matrix $W_{q}\in R^{d_{\mathrm{key}}\times d_{\mathrm{global}}}$. Overall, a single-head global attention layer uses three parameter matrices fit during training, $W_{q}$, $W_{k}$ and $W_{v}$. It is also parameterized by the key dimension $d_{\mathrm{key}}$ (we used $d_{\mathrm{key}}=64$). A multi-head global attention layer is obtained by applying $n_{\mathrm{heads}}$ independent single-head global attention layers (each with its own parameters) and concatenating their outputs, obtaining an output of dimension $n_{\mathrm{heads}}\cdot d_{\mathrm{value}}$ (we used $n_{\mathrm{heads}}=4$ across all 6 blocks of ProteinBERT). To satisfy dimensionality constraints, ProteinBERT uses $d_{\mathrm{value}}=\frac{d_{\mathrm{global}}}{n_{\mathrm{heads}}}=128$.

Overall, the ProteinBERT model includes six transformer-like blocks with four global attention heads in each block. Altogether, it includes ∼16M trainable parameters, making it substantially smaller than other protein language models. For comparison, there are ∼38M parameters in the TAPE Transformer (Rao *et al.*, 2019), ∼110M in BERT-base (Devlin *et al.*, 2018), ∼430M in ProtTrans's ProtBert-BFD (Elnaggar *et al.*, 2021), ∼650M in the ESM-1b model (Rives *et al.*, 2021) and 3B in ProtT5-XL-BFD (Elnaggar *et al.*, 2021).

The ProteinBERT architecture has several appealing properties. Most importantly, the entire architecture is agnostic to the length of the processed sequences, and it can be applied over sequences of any given length without changing its learned parameters (our experiments prove that the model indeed generalizes very well across different lengths). This good generality across sequence lengths is also achieved by avoiding positional embeddings used in the standard version of BERT which, in accordance with previous reports (Neishi and Yoshinaga, 2019) and our experimentation, do not always generalize well to sequence lengths longer than those present in the training data. Instead, the convolutional layers and special tokens used at the beginning and end of each sequence provide the model with information on the relative locations of positions. Due to the use of global attention rather than self-attention, the amount of computation performed by the model grows only linearly with sequence length (as opposed to quadratic growth in models with standard self-attention). This linear growth also applies to the model’s memory consumption, allowing ProteinBERT to process extremely long protein sequences (of tens of thousands of amino-acids) intact. Despite this simplification, each position in the local representations and sequence outputs can still depend on the content of each other position, thanks to the alternating information flow between the local and global representations. On top of that, the wide and narrow convolutional layers allow the representation of each position to depend on a large context. By relying on convolutional and attention layers, but avoiding recurrent layers, the computation performed by the network is more efficient and the learning is more stable with respect to sequence length (as there are no long-term dependencies and the computation performed by the network involves only a fixed number of tensor operations) (Hochreiter *et al.*, 2001). Notably, we did not use dropout or any other form of regularization (except for the final fully connected layer added when fine-tuning the model, which included dropout).

When fine-tuning ProteinBERT on a labeled dataset, another layer is added to its output. The final layer is fed with a concatenation of either the local or global hidden states of the model, depending on whether the output labels are local or global. The activation used for the final layer depends on the output type (i.e. softmax activation for categorical labels, sigmoid activation for binary labels or no activation for continuous labels).

### 2.6 Availability

Python code for ProteinBERT’s architecture, pretraining and fine-tuning is open source and available at https://github.com/nadavbra/protein_bert. The repository also includes pretrained model weights and code for downloading and generating the datasets and benchmarks. ProteinBERT is implemented in TensorFlow’s Keras (Abadi *et al.*, 2016; Chollet *et al.*, 2015).

## 3 Results

### 3.1 Pretraining improves protein modeling

**Fig. 2.**
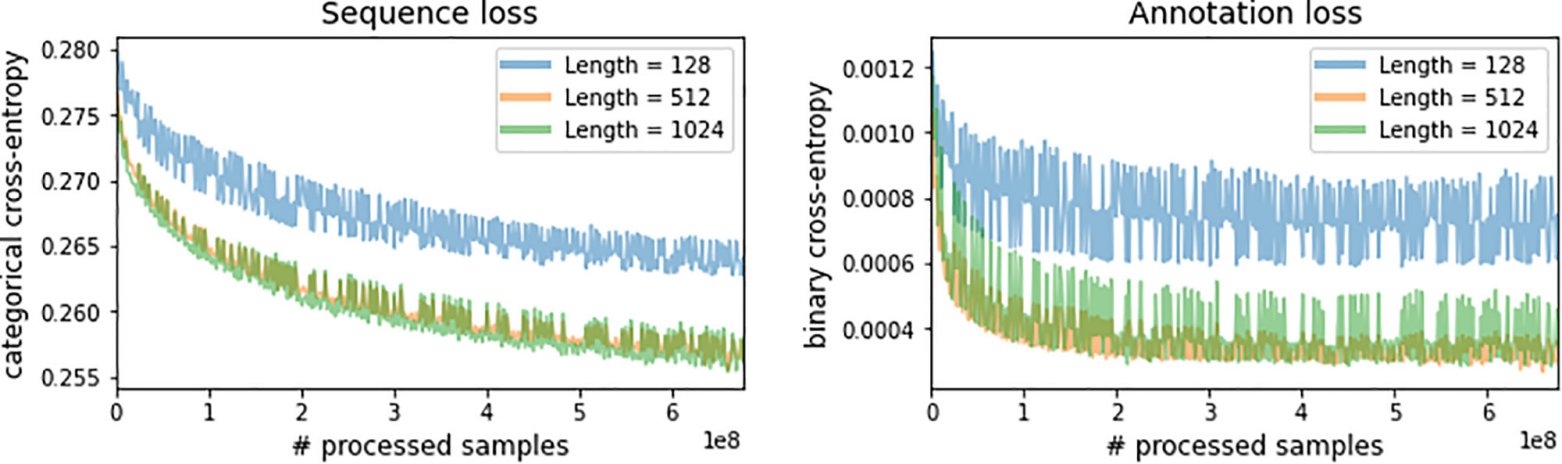
Pretraining loss. Training-set loss over the two pretraining tasks: (i) protein sequence language modeling, and (ii) GO annotation recovery. Losses were evaluated with input sequence length of 128, 512 or 1024 tokens on the first 100 batches of the dataset

ProteinBERT was pretrained on ∼106M UniRef90 records for ∼6.4 epochs. We see that the language modeling loss continues to improve on the training set (i.e. does not saturate), even after multiple epochs (Fig. 2), in accordance with other studies (Rives *et al.*, 2021). The GO annotations task, on the other hand, does show saturation. During pretraining, we periodically changed the sequence length used to encode the input and output protein sequences (128, 512 or 1024 tokens). We observe somewhat lower performance for the 128-token encoding, but similar for 512 and 1024.

### 3.2 ProteinBERT achieves nearly state-of-the-art results on diverse protein benchmarks

To evaluate ProteinBERT, we used nine benchmarks covering a variety of tasks in protein research (see definitions of the benchmarks in Table 1; full results for all benchmarks are available in Supplementary Table S1). For the four benchmarks taken from TAPE (secondary structure, remote homology, fluorescence and stability prediction), we compared our performance to other state-of-the-art sequence models which had been evaluated on the same benchmarks with the same metrics (Table 2). Specifically, we compared against a BERT Transformer and LSTM models evaluated in TAPE (Alley *et al.*, 2019; Bepler and Berger, 2019; Rao *et al.*, 2019). We also compared to ProtT5 (Elnaggar *et al.*, 2021) on the secondary-structure benchmark (which was the only one of the four TAPE benchmarks with published results for this model). Other notable protein language models [e.g. ESM (Rives *et al.*, 2021)] did not have directly comparable published results. Notably, the compared deep-learning models from TAPE have roughly 38M parameters and ProtT5-XL has 3 billion parameters, in contrast to ∼16M parameters in ProteinBERT. We evaluated ProteinBERT with and without pretraining, observing that pretraining has a major, positive effect on performance in many tasks. Across these benchmarks, ProteinBERT shows comparable performance, that sometimes exceeds similar, larger models trained with much more compute.

**Table 2.** TAPE benchmark results

	Method	Structure	Evolutionary	Engineering		Model size (M)
		Secondary structure - 3 state (accuracy)	Remote homology (accuracy)	Fluorescence (Spearman’s ρ)	Stability (Spearman’s ρ)	
Without Pretraining	TAPE Transformer	0.70	0.09	0.22	-0.06	38
	LSTM	0.71	0.12	0.21	0.28	~38^a^
	ProteinBERT	0.70	0.06	0.65	0.63	16
With Pretraining	TAPE Transformer	0.73	0.21	0.68	0.73	38
	LSTM	0.75	0.26	0.67	0.69	~38^a^
	UniRep mLSTM	0.73	0.23	0.67	0.73	18
	ProtT5-XL-BFD	0.77	–	–	–	3,000
	ProteinBERT	0.74	0.22	0.66	0.76	16

a Tape’s LSTM models “approximately match the number of parameters in the Transformer” (Rao *et al.,* 2019).

**Fig. 3.**
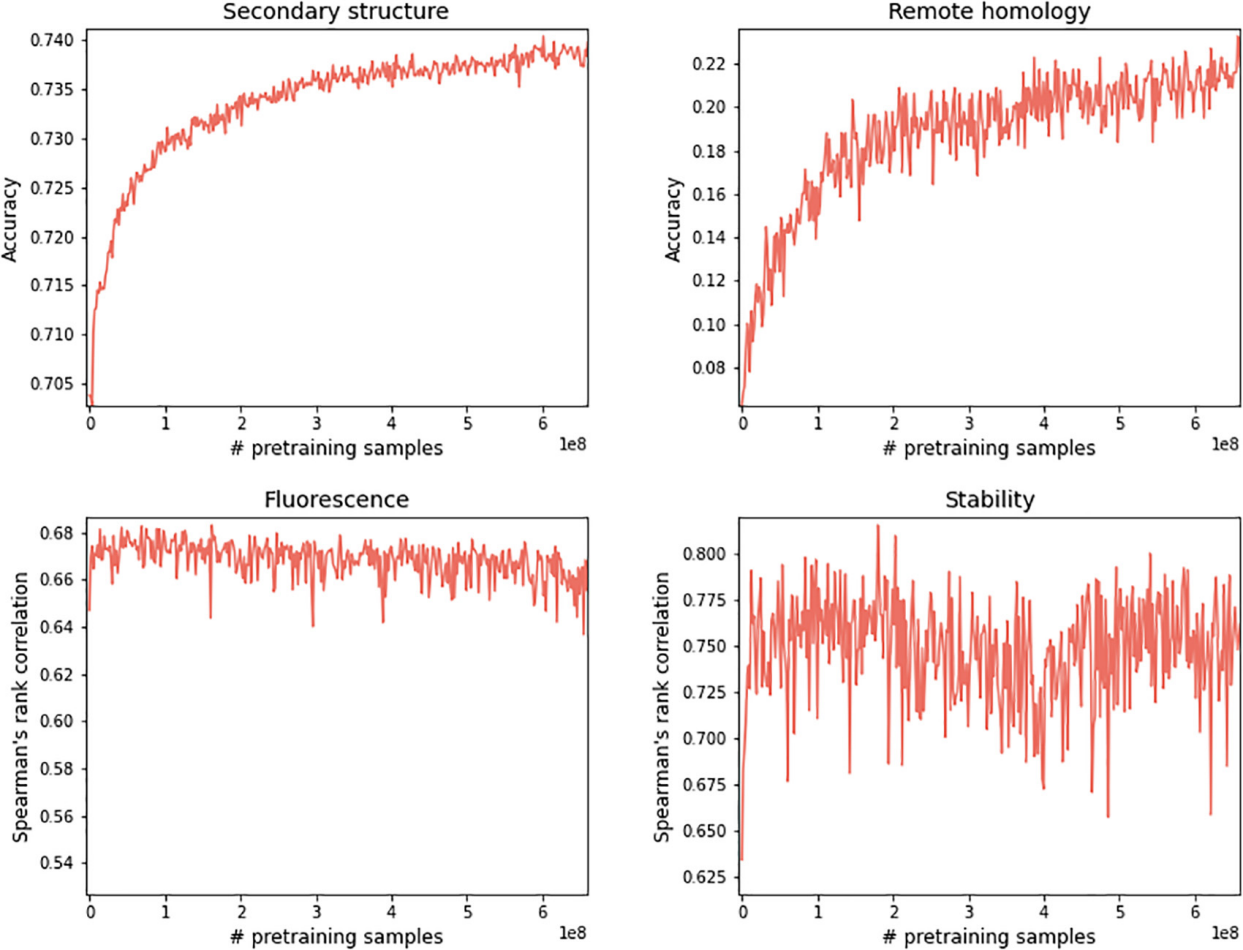
The impact of pretraining on downstream tasks. Performance of fine-tuned ProteinBERT models over the four TAPE benchmarks as a function of pretraining amount (measured by the number of processed proteins). Similar plots for all nine benchmarks are shown in Supplementary Figure S1

To further discern the impact of pretraining on downstream benchmark performance, we evaluated ProteinBERT following different pretraining durations. Specifically, we initiated the model from different snapshots along its pretraining and evaluated its down-stream performance after fine-tuning from these states (Fig. 3). While some tasks do not benefit from pretraining, other tasks (e.g. secondary structure and remote homology) show clear gains from ever more pretraining, and do not show saturation in that improvement. This is notable given that these are among the more challenging tasks.

We also performed ablation testing to study the effects of the GO-annotation pretraining task (Supplementary Fig. S2), and found that some benchmarks (specifically secondary structure, remote homology and fold classes) benefited from it.

### 3.3 ProteinBERT generalizes across protein lengths

**Fig. 4.**
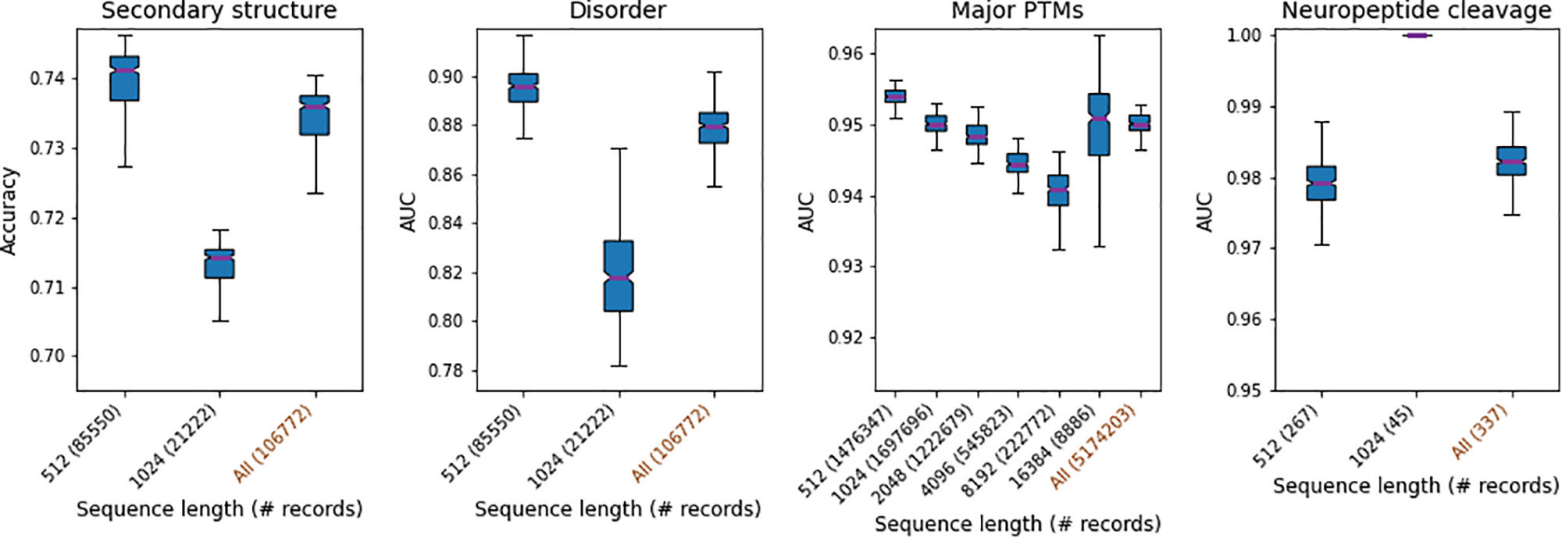
Performance across sequence lengths. Test-set performance of fine-tuned ProteinBERT models with different input sequence lengths. Sequence lengths (e.g. 512, 1024, etc.) always encode proteins of shorter lengths (e.g. a protein of 700 residues will be encoded as a 1024-long sequence). Boxplot distributions are over the 371 pretraining snapshots used in Figure 3

The architecture of ProteinBERT is efficient and flexible toward different sequence lengths (i.e. the number of tokens encoding the input and output sequences). To test the model’s capacity to generalize across sequence lengths, we measured the test-set performance of ProteinBERT on the 4 of 9 benchmarks that had a non-negligible number of test-set records in proteins longer than 512 tokens (Fig. 4). Specifically, we required at least 25 such records, where a record comprises either an entire protein (in the case of global tasks) or a residue (in the case of local tasks). We observe that in most cases ProteinBERT performs slightly worse for longer sequences, but only modestly, showing that it indeed generalizes across a very wide range of protein lengths. Moreover, the fact that in some cases longer sequences achieve better performance (e.g. 16 384-token sequences in the ‘Major PTMs’ benchmark, or 1024-token sequences in the ‘Neuropeptide cleavage’ benchmark) suggests that the changes in performance might be due to other factors (e.g. predicting the secondary structure of longer sequences might be an inherently more difficult task).

### 3.4 Understanding global attention

**Fig. 5.**
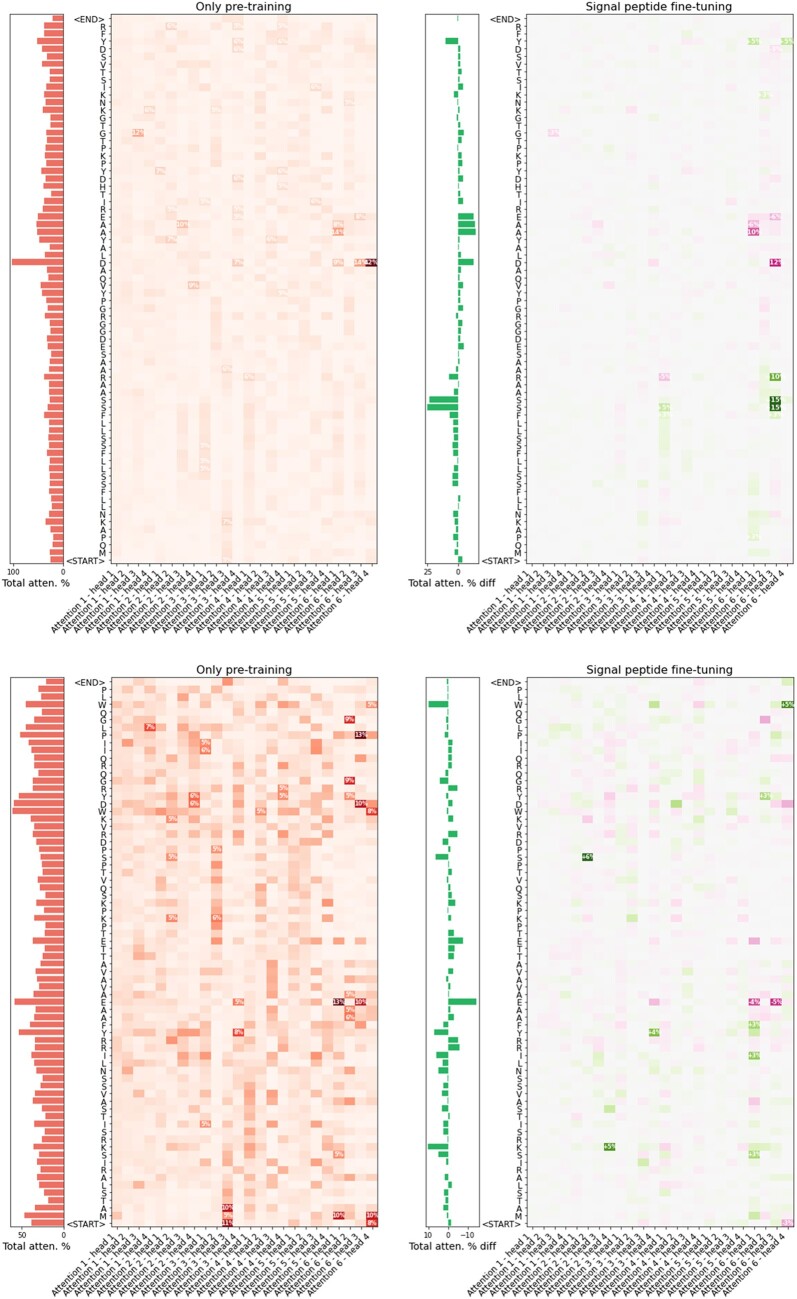
Global attention before and after fine-tuning on signal peptide prediction. Global attention values obtained for two selected proteins: Outer membrane protein P.IIC (piiC) in neisseria gonorrhoeae (top), and Gamma carbonic anhydrase-like 2, mitochondrial protein (GAMMACAL2) in arabidopsis (bottom). piiC has a signal peptide at positions 1–25 (ending with the amino-acid sequence SAARA). GAMMACAL2 has no signal peptide. The left panels (red colors) show the attention values obtained by the generic ProteinBERT model, after pretraining, it as a language model on UniRef90 (but before fine-tuning it on any specific task). The heatmap shows the global attention values at each residue of the protein by each of the 24 attention heads of the model. The bar plot shows the total attention at each residue by summing the attention values across all attention heads. The right panels show the difference in attention values after fine-tuning ProteinBERT on the signal peptide task. The heatmap shows the increase (green) or decrease (purple) of attention across all positions and attention heads. The bar plot shows the total difference in attention at each residue by summing the differences across all attention heads. Note that, each attention head necessarily sums up to 100%. Accordingly, differences sum up to 0%

To demonstrate the inner workings of the global attention mechanism, we extracted the values of the 24 attention heads in ProteinBERT for two unrelated proteins selected from the test-set of the signal peptide benchmark, before and after fine-tuning the model on that task (Fig. 5). The patterns of global attention are clearly distinct across different proteins, but some shared patterns exist. For example, attention head #3 in the 3rd block tends to concentrate on the beginning of protein sequences, while attention head #2 in the same layer tends to concentrate on the other parts. Fine-tuning the model on signal peptide prediction appears to have mostly altered the last (6th) global attention layer. For example, attention head #1 in that layer changed to further emphasize the beginning of sequences. In the positive example (Fig. 5, top panel), the largest increase in attention was at the end of the signal peptide (i.e. the cleavage site). It is worth stressing that the exact attention values are dependent on the model weights obtained from training, which can change between runs. From our experience, fine-tuning tends to produce rather consistent results, but small differences are sometimes observed.

## 4 Discussion

We have presented ProteinBERT, a new deep language model for protein sequences designed to capture local and global representations of proteins in a natural way (Fig. 1). We have demonstrated the universality of the model, showing that it can be fine-tuned on a wide range of protein tasks in a matter of minutes and achieve near state-of-the-art results (Table 2). While some larger protein language models [such as ProtT5 (Elnaggar *et al.*, 2021)] show better performance on at least some measured tasks, these models are far larger and involve orders-of-magnitude more compute and memory during both pretraining and inference.

ProteinBERT is extremely frugal by comparison to other leading protein language models with respect to size, compute and memory. For example, while ProteinBERT was pretrained for 4 weeks on a single GPU, UniRep was trained for 3.5 weeks on 4 GPUs (Alley *et al.*, 2019), and ProtTrans’s ProtT5-XL was trained on a supercomputer with thousands of GPUs and TPUs, and is too large to fit a single sequence on most consumer GPUs (Elnaggar *et al.*, 2021).

To pretrain ProteinBERT, we introduce a novel pretraining task of protein annotation prediction which is highly suited to proteins [unlike sentence order prediction and other natural language tasks (Lan *et al.*, 2019)]. We argue that GO annotations (Ashburner *et al.*, 2000) are a sensible extension to language modeling in proteins. They are ubiquitous and available for a large portion of curated proteins (∼46M of the ∼106M proteins in the UniRef90 dataset). In addition, they can teach the model about a wide range of protein functions (from subcellular localizations to pathways to biochemical roles).

Unlike previous works which included ∼250M putative, redundant sequences (Rives *et al.*, 2021), we constrained the pretraining of ProteinBERT to ∼106M representative proteins taken from UniRef90 (Suzek *et al.*, 2007), out of the entire known protein space of ∼215M proteins in UniProt (Boutet *et al.*, 2016). We argue that using a non-redundant set of proteins is more sensible and eliminates a lot of unnecessary bias caused by uneven sampling of the protein space, which is prevalent in the non-filtered version of UniProt. For example, there are >1M proteins in UniProt from the proteome of human immunodeficiency virus 1 (HIV-1), even though the real virus contains only 9 proteins. Such a redundancy reflects the abundance of sequence variations along HIV-1 evolution, and the great interest that researchers have had in this variation (compared with most other, far less studied organisms). Using a non-redundant set of proteins is also more efficient, especially when pretraining the model for less than an entire epoch (such as when searching for optimal hyper-parameter combinations).

Unlike traditional bioinformatic tools such as BLAST (Altschul *et al.*, 1990) and hidden Markov models (Finn *et al.*, 2014) which are based on sequence similarity (and therefore require searching through massive databases), the deep-learning approach taken in this work uses only primary sequence information, leading to two important advantages. First, it allows for rapid inference and dataset construction at a very large scale. Second, such models can remain effective in the presence of new sequences, whether or not they have homologues.

ProteinBERT’s architecture is efficient and highly scalable, allowing it to process protein sequences of any length. The same model weights conform to any sequence length, allowing it to be trained on a specific range of lengths and then generalize to other, unseen sequence lengths (Fig. 4). By supporting extremely long sequences (more than tens of thousands of residues), ProteinBERT spares the complication of splitting long sequences into smaller chunks, a common practice with self-attention-based models which grow quadratically (rather than linearly) with sequence length (Choromanski *et al.*, 2020; Zaheer *et al.*, 2020). At the core of the model’s flexibility is its use of global attention layers. The compactness of global attention (relative to self-attention) also allows easier inspection of the model’s attention, as all attention values (across all positions and attention heads) can be displayed as a simple 2D map (Fig. 5), as opposed to the 3D map that would be required to cover all-by-all self-attention.

Compatible with the general trends in the field of language modeling (Brown *et al.*, 2020), we observe that longer pretraining of ProteinBERT shows clear performance gains, both as a language model (Fig. 2) and across many specific tasks (Fig. 3, Supplementary Fig. S1). Existing works show that, other things being equal, larger models and additional pretraining correlates with improved model performance (Brown *et al.*, 2020; Devlin *et al.*, 2018; Rives *et al.*, 2021). Thus, we expect larger versions of ProteinBERT (e.g. with more, wider layers) to yield additional improvements. Yet, even with the frugal computing resources used in this work (a single GPU), ProteinBERT competes with state-of-the-art models (Table 2), providing a simple and efficient out-of-the-box solution for a wide range of protein tasks. The representations learned by the model through its pretraining are universally applicable across a wide array of tasks, making it useful for few-shot-learning tasks involving limited labeled data.

To facilitate easy usage of ProteinBERT, we provide the pretrained model as a Python package [based on TensorFlow and Keras (Abadi *et al.*, 2016; Chollet *et al.*, 2015)], which allows automatic downloading of a pretrained model state, fine-tuning and evaluation on labeled datasets, as well as scripts for creating the pretraining dataset.

By providing an effective and accessible model of protein sequence and function, we hope to expedite the adoption of deep language modeling by the protein research community and allow this new powerful tool to further push the boundaries of protein research.

## Supplementary Material

btac020_Supplementary_Data

## Data Availability

The data underlying this article are available at https://github.com/nadavbra/protein_bert.
